# Physical Exercise on Inflammatory Markers in Type 2 Diabetes Patients: A Systematic Review of Randomized Controlled Trials

**DOI:** 10.1155/2017/8523728

**Published:** 2017-03-19

**Authors:** Luciana Costa Melo, Jaime Dativo-Medeiros, Carlos Eduardo Menezes-Silva, Fabiano Timbó Barbosa, Célio Fernando de Sousa-Rodrigues, Luiza A. Rabelo

**Affiliations:** ^1^Programa de Pós-Graduação em Ciências da Saúde, Instituto de Ciências Biológicas e da Saúde, Universidade Federal de Alagoas, Campus A. C. Simões, Av. Lourival Melo Mota, s/n, Tabuleiro do Martins, 57072-900 Maceió, AL, Brazil; ^2^Laboratório de Reatividade Cardiovascular, Setor de Fisiologia, Núcleo de Síndrome Metabólica, Instituto de Ciências Biológicas e da Saúde, Universidade Federal de Alagoas, Maceió, AL, Brazil; ^3^Instituto Nacional de Ciência e Tecnologia em Nanobiofarmacêutica (N-BIOFAR), Belo Horizonte, MG, Brazil; ^4^Max Delbrück Center for Molecular Medicine, Berlin, Germany

## Abstract

*Background*. Type 2 diabetes mellitus (T2DM) is a serious disease associated with high morbidity and mortality. Scientific findings showed that physical exercise is an option for treatment of these patients. This study's objective is to investigate the effects of supervised aerobic and/or resistance physical training on inflammatory markers in subjects with T2DM.* Methods*. A systematic review was conducted on four databases, MEDLINE, CENTRAL, LILACS, and Scopus, and manual search from 21 to 30 November 2016. Randomized clinical trials involving individuals diagnosed with T2DM, who have undergone supervised training protocols, were selected in this study.* Results*. Eleven studies were included. Studies that evaluated control group versus aerobic exercise reported controversial results about the effectiveness of physical training in modifying C-reactive protein (CRP) and cytokine levels. The only variable analyzed by the six studies in comparison to the control group versus resistance exercise was CRP. This protein showed no significant difference between groups. Between the two modes of exercise (aerobic and resistance), only one study demonstrated that aerobic exercise was more effective in reducing CRP.* Conclusion*. The evidence was insufficient to prove that aerobic or resistance exercise improves systemic levels of inflammatory markers in patients with T2DM.

## 1. Introduction

The term diabetes mellitus (DM) describes a metabolic disorder of multiple etiologies characterized by chronic hyperglycemia, with carbohydrate, fat, and protein metabolism disorders resulting from defects in insulin secretion or action [1].

According to statistics, a DM epidemic is underway [2]. In 1985, an estimated 30 million adults around the world had diabetes; by 1995, this number had increased to 135 million, reaching 347 million in 2013, and diabetes is predicted to be the 7th leading cause of death by the year 2030 [3]. In this scenario, type 2 diabetes (T2DM) is the form present in 90% to 95% of cases [3].

Hyperglycemia, the main signal of DM, is even one component of the metabolic syndrome (SMet) which can be defined as the coexistence of metabolic disorders (abdominal obesity, hypertriglyceridemia, low HDL cholesterol, high blood pressure, or high fasting glucose) [4]. Although the metabolic pathways linking these disturbances are not completely clear, a proinflammatory state has been found to be an important element in the pathophysiology of the syndrome or T2DM [5, 6].

Obesity, especially visceral, is one of the most important factors in the development of diabetes through various mechanisms, such as increased circulating free fatty acids, adiponectin decrease, and secretion of cytokines in the adipose tissue, such as tumor necrosis factor-alpha (TNF-*α*) and interleukin-6. The proinflammatory molecules produced in the adipose tissue can activate pathways that result in disruption of systemic insulin sensitivity and glucose homeostasis that are characteristic of T2DM [5].

In addition to the participation of inflammatory processes in the pathogenesis of diabetes, evidence has shown that hyperglycemia itself contributes to the generation of proinflammatory factors. Hyperglycemia promotes the production of interleukin-1*β* (IL-1*β*) by pancreatic *β*-cells. IL-1*β* induces the production of various types of cytokines and chemokines through activation of the nuclear factor-*κ*B, which triggers the recruitment of macrophages [7]. In fact, cross-sectional and prospective studies have described elevated levels of C-reactive protein (CRP) [8], cytokines [8–10], and chemokines [8–10] in patients with T2DM.

With regard to diabetes treatment, physical exercise stands out as an important ally for glycemic control and other comorbid factors, such as hypertension and dyslipidemia, and for reducing cardiovascular risk [11, 12]. It is well established in the literature that physical exercise improves insulin sensitivity [13], increasing glucose uptake in muscles and adipocytes and reducing blood glucose levels [14]. Moreover, exercise increases blood glucose uptake by the muscles through insulin-independent mechanisms that involve GLUT4 activation by muscle contraction [15]. Thus, physical exercise facilitates glucose metabolism and its efficiency, improving glycemic control, which can be observed by the lower basal and postprandial insulin concentrations and the reduction of glycated hemoglobin in physically active diabetics when compared to sedentary patients [16, 17]. It is important to consider that the normalization of blood glucose is not sufficient to remove clinical outcomes in type 2 diabetes [17]. Despite the above-described scenario, to date, no reviews have demonstrated the effects of physical exercise on the inflammation characteristic of diabetes. Therefore, this study aims to investigate the effects of supervised aerobic and/or resistance physical training on inflammatory markers in T2DM patients. We ask for scientific evidence on randomized controlled trials in which patients with T2DM performed supervised exercise training protocols.

## 2. Methods

A protocol was drawn up in preparation for this review, which is available upon request to the corresponding author should there be a need for analysis. The study is reported according to the guidelines of the “Preferred Reporting Items for Systematic Reviews and Meta-Analyses” (PRISMA Statement; Supplementary Data available at https://doi.org/10.1155/2017/8523728) [18]. The results were not influenced by the source of the studies (authors or institutions where they were carried out). This systematic review was not registered with the International Prospective Register of Systematic Reviews (PROSPERO).

The search strategy aimed to identify randomized controlled trials (RCTs) that used physical exercise as a treatment strategy for patients with T2DM.

### 2.1. Databases

The studies were identified in four electronic databases: MEDLINE (via PubMed, from 2006 to November 2016), Cochrane Central Register of Controlled Trials (CENTRAL, Issues 3, 2016), Scopus (from 2006 to November 2016), and LILACS (via BIREME interface, 2006 to November 2016). The search procedure was carried out from 21 January to 26 November 2016.

The strategy used here considered the terms of most interest to the review: “Diabetes Mellitus,” “Exercise” and “Randomized controlled trial.” A combination of three terms were used in PubMed, Scopus, and LILACS: “Diabetes Mellitus” AND “Exercise” AND “Randomized controlled trial”, while a combination of two terms were used in CENTRAL: “Diabetes Mellitus” AND “Exercise”. In addition, searches were made in the reference lists of the selected articles for inclusion in this review. Text filters, availability, and/or origin of the study were not used. We used filters to publication date (articles published in the last 10 years) and age (studies with subjects aged 18 years or older).

### 2.2. Selection of Studies

RCTs were selected in which the participants were 18 years old or older, who had been definitively diagnosed with T2DM. The diagnosis was considered based on information contained in the article, regardless of the criteria used, without gender restriction.

The search focused on trials that used aerobic and/or resistance exercise in programs supervised by professionals. Trials in which the exercise program was associated with dietary or prescription medications to control T2DM were excluded. This could be a source of bias because the effects on the analyzed variables could be from these associated treatments.

We considered the control group as subjects that did not do supervised exercise program, maintained their daily routine activities, or were asked to perform less than 150 minutes of low grade exercise per week. We considered placebo training when subjects performed the same kind of exercise of trained groups but without load.

Trials composed of two groups performing different kinds of exercise (aerobic and resistance) were considered to compare the most effective exercise on modifying inflammatory biomarkers. On the other hand, studies comparing two groups of the same exercise on different intensities were not considered, because there are a large variety of intensities what could be a source of bias in the analysis.

Based on this selection, three possibilities were considered for analysis between groups: (i) aerobic exercise versus control, (ii) resistance exercise versus control, and (iii) aerobic exercise versus resistance exercise.

The first variable of interest was the plasma CRP level, because it is an inflammatory marker provenly altered in patients with T2DM [7]. Plasma cytokines associated with inflammation (adiponectin, tumor necrosis factor-*α*, interleukins, and/or other cytokines with specific activity) were considered secondary variables.

**Figure 1:**
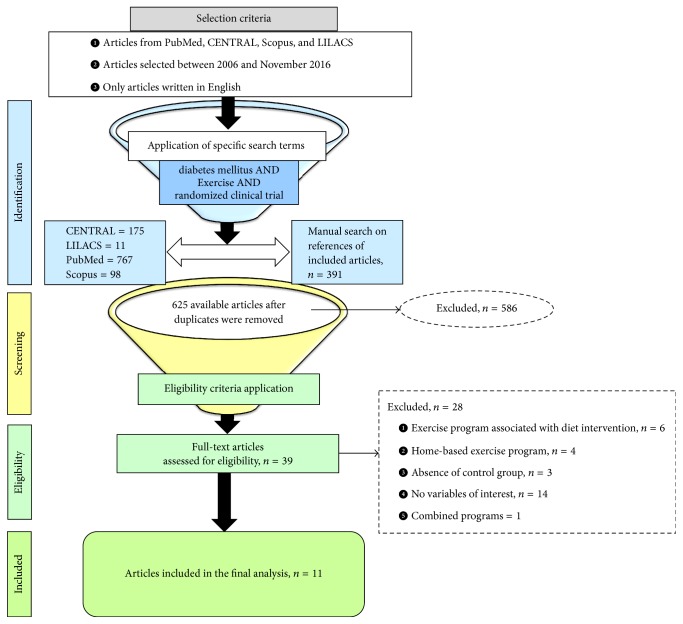
Workflow of information through the different phases of the systematic review.

The articles were identified by three authors independently, based on the titles and abstracts. Disagreements about their eligibility were resolved in a consensus meeting, after which the full texts of the selected articles were downloaded. The articles were then evaluated again independently by three authors of this review. The RCTs included in this review were defined by consensus.  Figure 1 summarizes the search methodology.

### 2.3. Extraction of Data

Preformatted forms were filled out containing identifying information about the study, characteristics of the sample, and data on the variables of interest. Data not available in the article were requested by email from the authors.

The data extraction procedure was performed by three authors independently. Each author read the included article and plotted the data of interest on tables previously designed. After this, the tables were compared to confirm accuracy of the extraction. Any disagreements about how to complete the forms were resolved in a consensus meeting.

Data from included studies were described as measures of central tendency and dispersion. Analysis was performed according to possible comparisons: (i) aerobic exercise versus control, (ii) resistance exercise versus control, and (iii) aerobic exercise versus resistance exercise.

### 2.4. Ethical Approval

This is a literature review-based study that did not involve experiments with animals or humans. No specific ethical approval is required for this kind of study and informed consent for participation is not applicable.

## 3. Results

### 3.1. Selection of Studies

The studies to be included in this review were identified during the period of 21 January 2016 to 26 November 2016. The search strategy resulted in the identification of 1,051 articles in the four aforementioned databases. After discarding duplicates, 625 articles were considered for the analysis of titles and abstracts. Among these, however, only 39 met the eligibility criteria and their full texts were analyzed. After analyzing the methodologies used in the randomized clinical trials, another 28 articles were excluded. Among these, six involved treatments associating physical exercise with dietary prescriptions, four involved unsupervised exercise programs, three compared two aerobic exercise programs at different levels of intensity but did not include a control group, one applied a protocol of aerobic exercise combined with resistance exercise, and fourteen did not evaluate CRP and/or inflammatory cytokine plasma levels. In the end, eleven studies met the preestablished eligibility criteria and were therefore included in this review. A manual search of the references listed in these seven articles was performed, but no studies were found that met the eligibility criteria besides the ones already selected (Figure 1).

### 3.2. Characteristics of Included Studies

The eleven studies selected to be included in this review were randomized controlled trials published in English, which used aerobic or resistance exercise for the treatment of patients above 18 years of age diagnosed with T2DM. Among these studies, four were conducted in Greece, two in the United States, one in New Zealand, one in Brazil, one in Australia, one in Iran, and one in Germany.

Among these eleven studies, ten used control groups. Two of these control groups received placebo training, three were instructed to perform 150 minutes per week of low-intensity physical activities, one was offered low-intensity stretching exercises once a week, and two were told to keep up their routine daily activities. Two studies did not describe the characteristics of their control groups.

Eight studies used aerobic training, while seven studies used resistance training. Considering the methods employed, it was possible to compare control group versus aerobic training, control group versus resistance training, and aerobic training versus resistance training in seven, six, and four studies, respectively (Table 1).  Table 2 describes the training programs and training intensities employed in these studies.

The seven studies included in this review involved 633 participants, all diagnosed with T2DM, who did not engage in regular physical activity. The plasma CRP levels were analyzed in ten studies (582 participants) before and after the full course of treatment.

Seven studies evaluated different inflammation-associated cytokines (Table 2): vaspin, human apelin-12, visfatin, ghrelin, adiponectin, resistin, tumor necrosis factor-*α* (TNF-*α*), interleukin-6 (IL-6), IL-10, and IL-18. All included studies were considered on descriptive analysis and discussion of the results.

### 3.3. Meta-Analysis

The design of this systematic review included a meta-analysis of plasma CRP and cytokine levels. However, upon reviewing the studies, it was impossible to perform this statistical procedure for the following reasons [19]:

1. Seven studies presented pre- and posttreatment analytical data [20–25], while four reported pre- and posttreatment delta variations [26–29] and three studies presented some results in a graph form, without providing data that would enable this analysis [23, 24, 26].
2. Eight studies presented their data as mean values ± standard deviations [22–27, 29, 30], one as mean ± standard error of the mean [21], another as median ± standard deviation [20], and one as mean and confidence interval [28].
3. The CRP data were expressed in different units of measure (mmol/l, mg/l, mg/l, pg/ml, and log).
4. The studies measured plasma levels of different cytokines. When more than one study analyzed the same cytokine, the above-described problems precluded the implementation of the meta-analysis.

The authors were asked via email to provide data required to perform the meta-analysis, but only one group [24] answered and sent the requested data.

After attempting to combine the studies to proceed with the meta-analysis, only two studies in each exercise modality were eligible for statistical analysis. This kind of analysis could not be able to translate the effect of interventions and was disconsidered.

### 3.4. Assessment of Heterogeneity

The sample of ten studies was composed of individuals of both sexes, albeit with a predominance of women. Moghadasi et al.'s (2013) [30] study had a sample composed of just men. The average age of the participants showed a little variation between the RCTs, with averages ranging from 48 to 68 years of age. On the other hand, the training programs varied significantly (Table 2). The duration of the exercise sessions was similar in seven studies (60 minutes). However, the frequency of training varied from 2 to 4 times per week. One of the studies did not report the duration and frequency of sessions, only the total workout time per week. Moreover, follow-up of the participants varied from three to twelve months.

The load applied in aerobic training sessions was defined based on different parameters: maximum heart rate, heart rate reserve, maximal oxygen uptake, and lactate threshold. Two studies did not report this load.

The intensity of resistance exercise was also variable: four studies used 60–80% of one maximum repetition, while two other studies considered the performance of a number of repetitions (10 or 12 repetitions) as the parameter of evaluation. One study did not describe the load applied in resistance training.

## 4. Discussion

The focus of this review was to investigate the effects of physical exercise on inflammatory markers. Analyzing the studies published until the selection procedure, we observed that the authors of the included RCTs used different training programs in their methodologies. Variations in time, frequency, and intensity of exercise impaired comparison between the studies. These are parameters that directly interfere in metabolic response to exercise [31–35] and comparisons between different treatments could be a source of bias. These facts prevented evidence from being gathered which would support or refute the hypothesis that physical exercise can improve the proinflammatory state of individuals with T2DM.

The studies that compare a group of individuals who performed aerobic exercise with a control group [21–23, 26, 28–30] present contradictory results about the effectiveness of training to reduce plasma CRP levels. While Kadaglou et al. [26], Oberbach et al. [21], and Kadaglou et al. [29] reported a reduction in the plasma CRP levels of trained individuals, three other studies found no significant difference in this parameter [22, 28, 30] when comparing the control group versus the aerobic group after the intervention. It was not possible to identify a specific outcome of exercise that could be related to different results from these studies. The aerobic training protocols used similar time of session training (30–60 minutes), but the divergence appears regarding duration of patient follow-up, weekly workout frequency, and training intensity.

As for the evaluation of cytokines, the data reported in the analyzed studies are still incipient. The two studies that analyzed TNF-*α* [22, 26] found no difference between the aerobic training group and the control group after the exercise protocol. There were no significant changes in adiponectin serum level in participants of two studies that evaluated this variable [22, 26]. On the other hand, adiponectin levels increased in the aerobic training group compared with pretraining values in Moghadasi et al.'s study [30]. Two other RCTs [23, 29] assessed ghrelin plasmatic level, but no significant changes were found between the groups compared.

Conflicting results were observed in the analysis of IL-6 and visfatin. Oberbach et al. [21] concluded that aerobic exercise reduces plasma levels of IL-6, while Jorge et al. [22] found no significant difference for this marker. Data of visfatin measurement demonstrated a reduction in participants of aerobic exercise program in comparison with controls [29]. On the other hand, Jorge et al. [22] found an increase in visfatin serum level in all groups, including control, after the follow-up period when compared with baseline measurements.

Increments on human apelin-12 serum level in the aerobic exercise group rather than in the control groups were found in two studies [23, 29], but both from the same population. Only one study evaluated serum level of resistin [22], vaspin [29], IL-18 [26], and IL-10 [26].

In studies comparing individuals in resistance training with a control group [20, 22, 25, 27–29], the only variable of interest analyzed for all was CRP. Four studies reported finding no significant difference between the control and trained groups [22, 27–29] while two trials found a reduction in CRP serum level in resistance training groups when compared with the control group [20, 25]. Visfatin serum level was analyzed in two studies [19, 22]; in both, there were no significant changes after resistance exercise program when compared with the control group. Brooks et al. [20] reported reduction in adiponectin serum levels in the treated group compared with the control group while Kadoglou et al. [27] found no significant difference. Vaspin [29], human apelin-12 [29], ghrelin [29], resistin [22], TNF-*α* [22], and interleukin-6 [22] were evaluated in just one of the six studies. This makes conclusive analysis impossible. Furthermore, such findings should be considered carefully because when resistance loads, weekly training frequency, and follow-up periods differ, there can be no reproducibility of results.

Among the studies that compared the two types of exercise (aerobic and resistance) [22, 24, 28, 29], only Kadaglou et al. [29] demonstrated that aerobic exercise decreased the CRP levels in comparison to those individuals undergoing resistance training. Also, in this study, an increment in apelin and vaspin was found after aerobic training in comparison with the resistance group. The studies found no difference in the levels of other cytokines (ghrelin [22, 29], adiponectin [22, 24], TNF-*α* [22], resistin [22], and IL-6 [22]) between the groups after the training protocol.

It is important to highlight that T2DM is a disease with multifactorial etiology that affects many physiological systems. Because of this, the treatment must contemplate several strategies in order to promote the health of individuals with T2DM. Data from the Italian Diabetes and Exercise Study (IDES) [36–38] showed that aerobic or combined (aerobic and resistance) exercise was effective in reducing CRP plasma levels after 12 months of training in T2DM patients receiving standard medical care (nutritional guidance and pharmacological treatment). Similar results were observed in other inflammatory markers. Leptin, resistin, and interleukin-6 decreased, whereas adiponectin increased in exercising groups [37]. Interleukin-1*β*, tumor necrosis factor-*α*, and interferon-*γ* decreased, whereas anti-inflammatory interleukin-4 and interleukin-10 increased only in the group that performed combined exercise [37].

On the other hand, even in studies with other treatments associated with exercise, the results are contradictory. While Choi et al. [39] demonstrated that CRP decreased in an exercising group of T2DM patients after 12 weeks, no significant differences were observed in interleukin-6. Byrkjeland et al. [40] found no difference in CRP plasma level from individuals in a training program or sedentary individuals.

It is interesting that changes in VO_2_max, exercise modalities, and training supervision were strong predictors of inflammation reduction in IDES [36–38]. In the analyzed studies in this review, it is possible that divergences in results are related to characteristics of applied exercise. This reinforces the importance of individuality in the prescription of exercise program and the supervision of training by a professional.

In this review, two RCTs did not clearly state the intensity of aerobic exercise [21] and the load applied in resistance training [22]. This seriously compromises the systematic analysis of results, since training intensity implies different metabolic alterations [33, 34]. Thus, the results of studies that used different training modalities and/or intensities cannot be compared.

Although this systematic review identified a sufficient number of RCTs to perform a meta-analysis, their methodological heterogeneity led to a careful analysis in terms of clinical applicability of the results, since they are from different interventions.

The presentation of data in different units of measure or in graph format (without numerical information on mean and standard deviation) prevented a statistical comparison of the results of most included studies. This showed us that there is no standardization in the presentation format of the biochemical data in the analyzed scientific articles, which makes an analysis to provide subsidies to refute or confirm the initial hypotheses of this systematic review difficult.

From the standpoint of clinical applicability, the studies show little power to endorse the recommendation of physical exercise as a strategy to reverse or control the inflammatory state in diabetes. However, previously published reviews have shown that exercise is effective for glycemic control [34, 35] and improved arterial function [41]. Therefore, the results of this review do not contraindicate the use of exercise in the nonpharmacological therapeutic management of diabetes, since there is evidence of benefits for other disorders presented by individuals with T2DM [34, 35, 41].

Limitations of this study were the presence of methodological heterogeneity in the studies selected for this meta-analysis, the lack of data that could be analyzed by meta-analysis, and the restriction of the selection to studies on T2DM.

## 5. Conclusions

The evidence was insufficient to prove that aerobic or resistance training improves plasma levels of inflammatory markers in patients with T2DM. Thus, we recommend further studies designed with the methodological rigor necessary to prove or disprove the effectiveness of this strategy in reversing inflammation associated with T2DM.

To date, no other reviews have been published about the effects of physical exercise on inflammation in patients with T2DM. Thus, the systematic review of scientific literature on the subject in question provides supporting evidence to underpin new studies that can meet the methodological requirements for the establishment of scientific evidence.

## Supplementary Material

The studies were identified in four electronic databases – MEDLINE, CENTRAL, SCOPUS and LILACS from 21 January to 26 November 2016. The strategy used here considered the terms of most interest to the review: “Diabetes Mellitus,” “Exercise” and “Randomized controlled trial.” Randomized Clinical Trials RTCs were selected in which the participants were 18 years or older and who had been definitively diagnosed with T2DM. Three authors identified the articles independently, based on the titles and abstracts. Disagreements about their eligibility were resolved in a consensus meeting, after which the full texts of the selected articles were downloaded and three authors of this review evaluated the articles again. The RTCs included in this review were defined by consensus.

## Figures and Tables

**Table 1:** Treatments compared in the included studies.

Control group versus aerobic training	Control group versus resistance training	Aerobic training versus resistance training
Kadoglou et al., 2007 [26] Oberbach et al., 2008 [21] Jorge et al., 2011 [22] Swift et al., 2012 [28] Kadoglou et al., 2012 [23] Kadoglou et al., 2013 [29] Moghadasi et al., 2013 [30]	Brooks et al., 2007 [20] Jorge et al., 2011 [22] Swift et al., 2012 [28] Kadoglou et al., 2012 [27] Kadoglou et al., 2013 [29] Mavros et al., 2014 [25]	Jorge et al., 2011 [22] Sukala et al., 2012 [24] Swift et al., 2012 [28] Kadoglou et al., 2013 [29]

**Table 2:** Description of training programs employed in the treatment of participants in randomized clinical trials.

Author, year	Country	Participants	Interventions, group (*n*)	Intervention period	Inflammatory status variables analyzed	Results	Remarks
Brooks et al., 2007 [20]	United States	Hispanic man and woman, 55 years old and older with diagnosed T2DM	*Control (31)*: recommended to continue usual standard of care. *Resistance (31)*: 60–80% of 1RM during the first 9 weeks and 70–80% of 1RM on weeks 10–16.	45 min/session, 3x/week, for 4 months	CRP (mg/l) Adiponectin (*µ*g/ml)	Reduction in serum CRP level in resistance training group compared with control group. Increase in adiponectin concentrations in resistance group compared with control group.	Data from CRP and adiponectin were exposed as median and interquartile range.

Kadoglou et al., 2007 [26]	Greece	Man and woman with diagnosed T2DM	*Control (30)*: no programmed activity. *Aerobic (30)*: 50–75% of maximum O_2_ consumption.	60 min/session, 4x/week, for 6 months	hsCRP (mg/dl) Adiponectin (*μ*g/ml) TNF-*α* (pg/ml) Interleukin-18 (pg/ml) Interleukin-10 (pg/ml)	Reduction in hsCRP and interleukin-18 after aerobic training compared with control group. Increase in interleukin-10 after aerobic training compared with control group. Nonsignificant changes in TNF-*α* or adiponectin in either group or between the groups after the intervention.	Data from hsCRP, adiponectin, TNF-*α*, interleukin-10, and interleukin-18 were exposed on graphs without values of mean and standard deviation.

Oberbach et al., 2008 [21]	Germany	Man and woman with glucose impaired tolerance	*Control (16)*: not described. *Aerobic (24)*: load not described.	60 min/session, 2x/week, for 12 months	hsCRP (pg/ml) Interleukin-6 (pg/ml)	Reduction in hsCRP and interleukin-6 serum level in the aerobic group after intervention.	Data before and after intervention exposed on analytical values (mean ± standard error of mean).

Jorge et al., 2011 [22]	Brazil	Man and woman aged 30–70 years diagnosed with T2DM and with body mass index between 25 and 40 kg/m^2^	*Control (12)*: light stretching exercise. *Aerobic (12)*: up to lactate threshold. *Resistance (12)*: load not described. *Combination (12)*: aerobic and resistance training with the same intensity of the other training groups and half of the exercise volume on each category.	60 min/session, 3x/week, for 3 months	hsCRP (mg/ml) Adiponectin (*μ*g/ml) Visfatin (ng/ml) Resistin (ng/ml) TNF-*α* (pg/ml) Interleukin -6 (pg/ml)	Reduction in hsCRP serum level in all groups, including control after intervention. Increase in visfatin serum level in all groups, including control after intervention. Nonsignificant changes in adiponectin, resistin, TNF-*α*, or interleukin-6 in either group after intervention.	Data before and after intervention exposed on analytical values (mean ± standard deviation).

Kadoglou et al., 2012 [23]	Greece	Man and woman aged 50–70 years with a diagnosis of T2DM	*Control (27)*: 150 minutes/week of self-managed physical activity. *Aerobic (26)*: progressive load until 60%–75% of HR_max_.	60 min/session, 4x/week, for 3 months	Human apelin-12 (ng/ml) Ghrelin (ng/ml)	Increment on apelin serum level after the intervention period on aerobic training group compared with control group. Nonsignificant effect on ghrelin serum level in the intervention group.	Data from apelin and ghrelin were exposed on graphs without values of mean and standard deviation.

Kadoglou et al., 2012 [27]	Greece	Man and woman with T2DM diagnosed for more than 1 year	*Control (24)*: 150 minutes/week of self-managed physical activity. *Resistance (23)*: 2-3 sets of 6–8 repetitions on machine weights. Exercise intensity of 60–80% of 1RM.	60 min/session, 3x/week, for 3 months	hsCRP (mg/l)	Nonsignificant changes in CRP levels between the groups after the intervention.	Data expressed as delta variation (mean ± standard deviation).

Sukala et al., 2012 [24]	New Zealand	Man and woman self-identified Polynesian descent, with clinical diagnosis of T2DM and visceral obesity	*Aerobic (9)*: 65–80% of heart rate reserve (HR_reserve_). *Resistance (9)*: progressive load (5%) starting from the execution of 10 repetitions.	40 to 60 min/session, 3x/week, for 4 months	CRP (log) Adiponectin (*μ*g/ml)	Nonsignificant changes in CRP or adiponectin in either group or between the groups after the intervention.	Data expressed as delta variation (mean ± standard deviation).

Swift et al., 2012 [28]	United States	Man and woman aged 35–70 years with diagnosed T2DM	*Control (37)*: weekly stretching and relaxation. *Aerobic (50)*: 50–80% of maximum O_2_ consumption. *Resistance (58)*: progressive load starting from the execution of 12 repetitions. *Combination (59)*: combination of aerobic training (10 kcal/kg/week) plus 2 sessions of resistance training per week.	150 min/week, 3x/week, for 9 months	CRP (mg/l)	Nonsignificant change in CRP serum level in either group or between the groups after the intervention.	Data expressed as delta variation (mean ± confidence interval).

Kadoglou et al., 2013 [29]	Greece	Man and woman with T2DM diagnosed for more than 1 year.	*Control (24)*: 150 minutes/week of self-managed physical activity. *Aerobic (21)*: progressive load until 60%–75% of HR_max_. *Resistance (23)*: load of 60%–80% of 1-MR. *Combination (22)*: aerobic and resistance training with the same intensity of the other training groups and half of the exercise volume on each category.	60 min/session, 4x/week, for 6 months	hsCRP (mmol/l) Vaspin (ng/ml) Human apelin-12 (ng/ml) Visfatin (ng/ml) Ghrelin (ng/ml)	Reduction in hsCRP and visfatin serum level after aerobic and combined training compared with both resistance training alone and control groups. Increment on apelin and vaspin serum level in the aerobic exercise and combined groups rather than in the control and resistance training groups. Nonsignificant effect on ghrelin serum level in the intervention groups.	Data from vaspin, apelin, visfatin, and ghrelin were exposed on graphs without values of mean and standard deviation.

Moghadasi et al., 2013 [30]	Iran	Obese and overweight man with T2DM	*Control (8)*: not described. *Aerobic (8)*: 40–59% of maximum O_2_ consumption.	30 min/session, 4x/week, for 3 months	hsCRP (mg/ml) Adiponectin (*µ*g/l)	Nonsignificant changes in CRP in either group or between the groups after the intervention. Adiponectin level increased in training group compared with pretraining values.	—

Mavros et al., 2014 [25]	Australia	Man and woman ≥60 years of age, sedentary with type 2 diabetes and metabolic syndrome	*Control (47)*: training on the same equipment used by resistance group, 3 times a week, under supervision from the same trainers. Exercise intensity as low as possible and not progressing. *Resistance (41)*: 3 sets of 8 repetitions. Exercise intensity of 80% of 1RM.	Session duration not informed, 3x/week, for 12 months	CRP (mg/l)	Reduction in CRP in resistance group relative to control group.	—

## Abbreviations

1-MR: One maximum repetition
CRP: C-reactive protein
DM: Diabetes mellitus
GLUT4: Glucose transporter 4
HDL: High-density lipoprotein
HR_max_: Maximum heart rate
IL: Interleukin
PRISMA: Preferred Reporting Items for Systematic Reviews and Meta-Analyses
RCT: Randomized controlled trial
T2DM: Type 2 diabetes mellitus
TNF-*α*: Tumor necrosis factor-alpha.
