# Supplementary figures and images for: Physical Exercise on Inflammatory Markers in Type 2 Diabetes Patients: A Systematic Review of Randomized Controlled Trials
Source: Oxid Med Cell Longev. 2017 Mar 19;2017:8523728. doi: 10.1155/2017/8523728 (PMC5376457; doi:10.1155/2017/8523728)

# Flow Diagram – PRISMA Statement

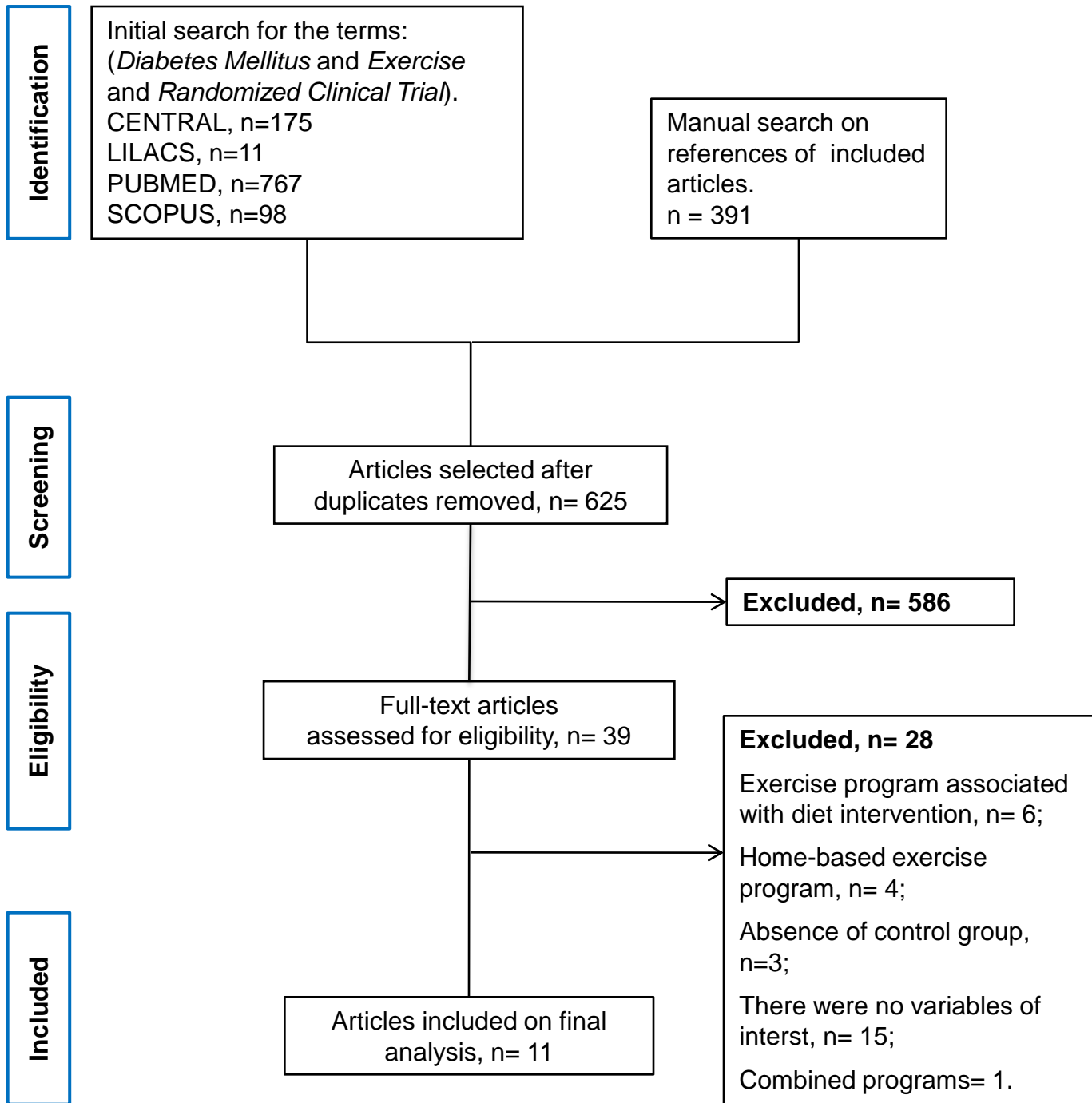

Supplement: Supplementary file 1 — The studies were identified in four electronic databases – MEDLINE, CENTRAL, SCOPUS and LILACS from 21 January to 26 November 2016. The strategy used here considered the terms of most interest to the review: “Diabetes Mellitus,” “Exercise” and “Randomized controlled trial.” Randomized Clinical Trials RTCs were selected in which the participants were 18 years or older and who had been definitively diagnosed with T2DM. Three authors identified the articles independently, based on the titles and abstracts. Disagreements about their eligibility were resolved in a consensus meeting, after which the full texts of the selected articles were downloaded and three authors of this review evaluated the articles again. The RTCs included in this review were defined by consensus. [file 8523728.f1.pdf]
